# Retinal and Choroidal Thickness in an Indigenous Population from Ghana: Comparison with Individuals with European or African Ancestry

**DOI:** 10.1016/j.xops.2023.100386

**Published:** 2023-08-21

**Authors:** Moussa A. Zouache, Caitlin D. Faust, Vittorio Silvestri, Stephen Akafo, Seth Lartey, Rajnikant Mehta, Joseph Carroll, Giuliana Silvestri, Gregory S. Hageman, Winfried M. Amoaku

**Affiliations:** 1 Sharon Eccles Steele Center for Translational Medicine, John A. Moran Eye Center, Department of Ophthalmology & Visual Sciences, University of Utah, Salt Lake City, Utah; 2 NICRN, Belfast Health and Social Care Trust, Belfast, UK; 3 Unit of Ophthalmology, Department of Surgery, University of Ghana Medical School, Accra, Ghana; 4 Eye Unit, Eye Ear Nose and Throat Department, Komfo Anokye Teaching Hospital and Kwame Nkrumah University of Science and Technology, Kumasi, Ghana; 5 Research Design Service, East Midlands (RDS EM), University of Nottingham, Nottingham, UK; 6 Department of Ophthalmology & Visual Sciences, Medical College of Wisconsin Eye Institute, Milwaukee, Wisconsin; 7 Ophthalmology Department, Belfast Health and Social Care Trust, Belfast, UK; 8 Academic Ophthalmology and Visual Sciences, Mental Health & Clinical Neurosciences (Academic Unit 1), University Hospital, QMC, Nottingham, UK

**Keywords:** Africans, African-Americans, Choroid, OCT

## Abstract

**Purpose:**

To evaluate the thickness of the macular retina and central choroid in an indigenous population from Ghana, Africa and to compare them with those measured among individuals with European or African ancestry.

**Design:**

Cross-sectional study, systematic review, and meta-analyses.

**Participants:**

Forty-two healthy Ghanaians, 37 healthy individuals with European ancestry, and an additional 1427 healthy subjects with African ancestry from previously published studies.

**Methods:**

Macular retinal thickness in the fovea, parafovea, and perifovea and central choroidal thickness were extracted from OCT volume scans. Associations with ethnicity, age, and sex were assessed using mixed-effect regression models. Monte Carlo simulations were performed to determine the sensitivity of significant associations to additional potential confounders. Pooled estimates of retinal thickness among other groups with African ancestry were generated through systematic review and meta-analyses.

**Main Outcome Measures:**

Macular retinal thickness and central choroidal thickness and their association with ethnicity, age, and sex.

**Results:**

When adjusted for age and sex, the macular retina and central choroid of Ghanaians are significantly thinner as compared with subjects with European ancestry (*P* < 0.001). A reduction in retinal and choroidal thickness is observed with age, although this effect is independent of ethnicity. Meta-analyses indicate that retinal thickness among Ghanaians differs markedly from that of African Americans and other previously reported indigenous African populations.

**Conclusions:**

The thickness of the retina among Ghanaians differs not only from those measured among individuals with European ancestry, but also from those obtained from African Americans. Normative retinal and choroidal parameters determined among individuals with African or European ancestry may not be sufficient to describe indigenous African populations.

**Financial Disclosure(s):**

Proprietary or commercial disclosure may be found in the Footnotes and Disclosures at the end of this article.

Despite possessing more genetic variation than any other region,^1^, ^2^, ^3^ Africa is under-represented in scientific studies,^4^^,^^5^ including in ophthalmology.^6^ Because of this, the way in which genetic and environmental variation within the continent and divergence from groups from other geographical locations (such as Europe or Asia) influence the prevalence and/or clinical presentation of ocular diseases among indigenous African populations is poorly characterized.^7^ Evidence points towards significant differences between individuals of African and European descent. African Americans are 15 times more likely to develop glaucoma, the second leading cause of blindness worldwide, and are 7 times more likely to suffer permanent vision loss from it.^8^^,^^9^ They are on average 4 times more likely to be afflicted by diabetic retinopathy.^10^^,^^11^ Retinal vein occlusion is more common among African Americans than individuals with European ancestry.^12^ The early stage of age-related macular degeneration (AMD) affects a larger proportion of Europeans (11.2%) than Africans (7.1%).^13^ The late stages of the disease are almost twice as prevalent amongst Europeans as compared to Africans (0.5% vs. 0.3%, respectively).^13^ Phenotypically, drusen and focal hyperpigmentation, 2 features that are characteristic of early and intermediate AMD, are more common amongst Europeans compared with individuals with African ancestry.^14^^,^^15^ Non-neovascular AMD features such as geographic atrophy are more common outside the macula among African Americans patients.^15^ Certain diseases of the choroid such as polypoidal choroidal vasculopathy have clear ethnic predispositions among individuals with African ancestry.^16^^,^^17^ While presumed to be more common in European populations, central serous chorioretinopathy appears to be more aggressive among African American patients.^18^

While valuable, these observations are limited by the fact that subjects of African descent considered in these studies only account for a small proportion of the genetic diversity characteristic of Africa. For instance, the ancestry of African Americans is predominantly from the Niger-Kordofanian region (∼71%) and European populations (∼13%), with levels of admixture varying considerably between individuals.^3^ Additional determinants of visual impairments including environmental and socioeconomic factors such as education level, income, employment, and distance to care facilities also contribute to the variability in susceptibility to ocular diseases and are likely to vary between African, African American, and European populations.^7^^,^^19^, ^20^, ^21^ Findings established among African Americans and individuals of European descent may therefore not be entirely translatable to indigenous African populations. Considering this, it is necessary to establish normative comparisons between indigenous African populations and individuals of African and European ancestry. Systematic comparisons between these different groups would ensure that biomarkers specific to indigenous populations may be identified, so that disease risk and prognosis may be accurately assessed. This is a decisive step toward improved clinical care and precision medicine.^22^

This study was undertaken to evaluate macular retinal thickness and central choroidal thickness in a healthy indigenous African population from Ghana, and to compare them with those measured among individuals with European ancestry and previously reported populations of African descent. The thickness of the retina and choroid are commonly used to diagnose, monitor, and estimate the risk of progression of many vitreoretinal disorders.^23^, ^24^, ^25^, ^26^, ^27^, ^28^ These measures rely on OCT, an established and widely available imaging technique that allows for 3-dimensional reconstructions of regions of the macula and posterior pole. Existing databases for normative OCT measurements, including retinal and choroidal thickness, have mostly been established using individuals with European descent and often overlook baseline differences in retinal and choroidal anatomy between ethnic groups.^29^ Previous studies have, however, shown that ethnicity influenced retinal and choroidal thickness significantly, including among African Americans^30^, ^31^, ^32^, ^33^, ^34^, ^35^, ^36^ and Afro-Caribbeans.^37^^,^^38^ Indigenous African populations without ocular diseases have been considered in a limited number of studies only,^34^^,^^39^^,^^40^ with macular retinal thickness being the main feature examined. Ghanaians included in this study were recruited from 2 locations, the capital Accra and Kumasi in the Ashanti region. While the fine-scale genetic structure within west Africa is complex, previous studies indicate that these participants have a close genetic relationship within the Niger-Congo area, which is part of the larger Niger-Kordofanian region.^41^^,^^42^

## Methods

### Subjects

Ghanaian subjects were recruited as part of a prospective study carried out at 2 teaching hospitals in Ghana, the Korle-Bu Hospital in Accra and the Komfo Anokye Teaching Hospital in Kumasi. The aims and details of the study were explained to all subjects with the help of interpreters (if necessary) and information leaflets, and written consent was obtained from all patients. A questionnaire detailing the Ghanaian patients’ ophthalmic and medical histories was completed by the local ophthalmic nursing staff. Best corrected Snellen visual acuity corrected by glasses or pinhole was assessed for all participants. Individuals with self-reported European ancestry were recruited from local communities surrounding the Medical College of Wisconsin (Milwaukee, WI).^43^ To ensure consistency in age representation between the Ghanaian and European groups, all Ghanaians participants younger than 40 (2 subjects) were excluded from analyses. The research adhered to the tenets of the Declaration of Helsinki and the protocol was approved by the ethics review boards of both the Korle-Bu Hospital (University of Ghana Medical School Protocol ID No: MS-Et/M.4-P./2008/2009) and the Komfo Anokye Teaching Hospital (KNUST Reference: CHRPE/32/10) for Ghanaian participants and by the institutional review board at the Medical College of Wisconsin for individuals with European ancestry.

### Imaging and Screening for Retinal Diseases

The same imaging protocol was applied for all Ghanaians and participants of European descent. All patients were dilated prior to imaging using tropicamide 1% and phenylephrine 2.5%. Each patient underwent a slit lamp biomicroscopy and fundus examination performed by a retina specialist in addition to an OCT scan in both eyes when possible. A Zeiss Cirrus high-definition (HD)-OCT (Carl Zeiss Meditec) was employed for capture in all study sites. This device does not rely on the patient’s ability to fixate, and it allows for post-hoc repositioning of the scan to the center of the fovea. Volumes acquired in all participants were nominally 6 × 6 mm and consisted of ≥ 128 B-scans (512 A-scans/B-scans). The signal strength for all scans was ≥ 7.^44^^,^^45^ Screening of the images for vitreoretinal and choroidal disorders was performed by G.S., W.A., and G.S.H. using fundus and OCT images. Eyes presenting with a history of vision-limiting ocular diseases or clinical marker of any vitreoretinal disorder including AMD, diabetic retinopathy, retinal vascular diseases, epiretinal membranes, vitreomacular traction, intraretinal cysts, hereditary macular disease, or macular holes were excluded. Eyes with myopia of more than −4.00 diopters (D) were also excluded from these analyses. Axial length (AL) was measured among subjects with European ancestry only using the IOLMaster ocular biometer (Carl Zeiss Meditec).

### Image Segmentation

**Figure 1:**
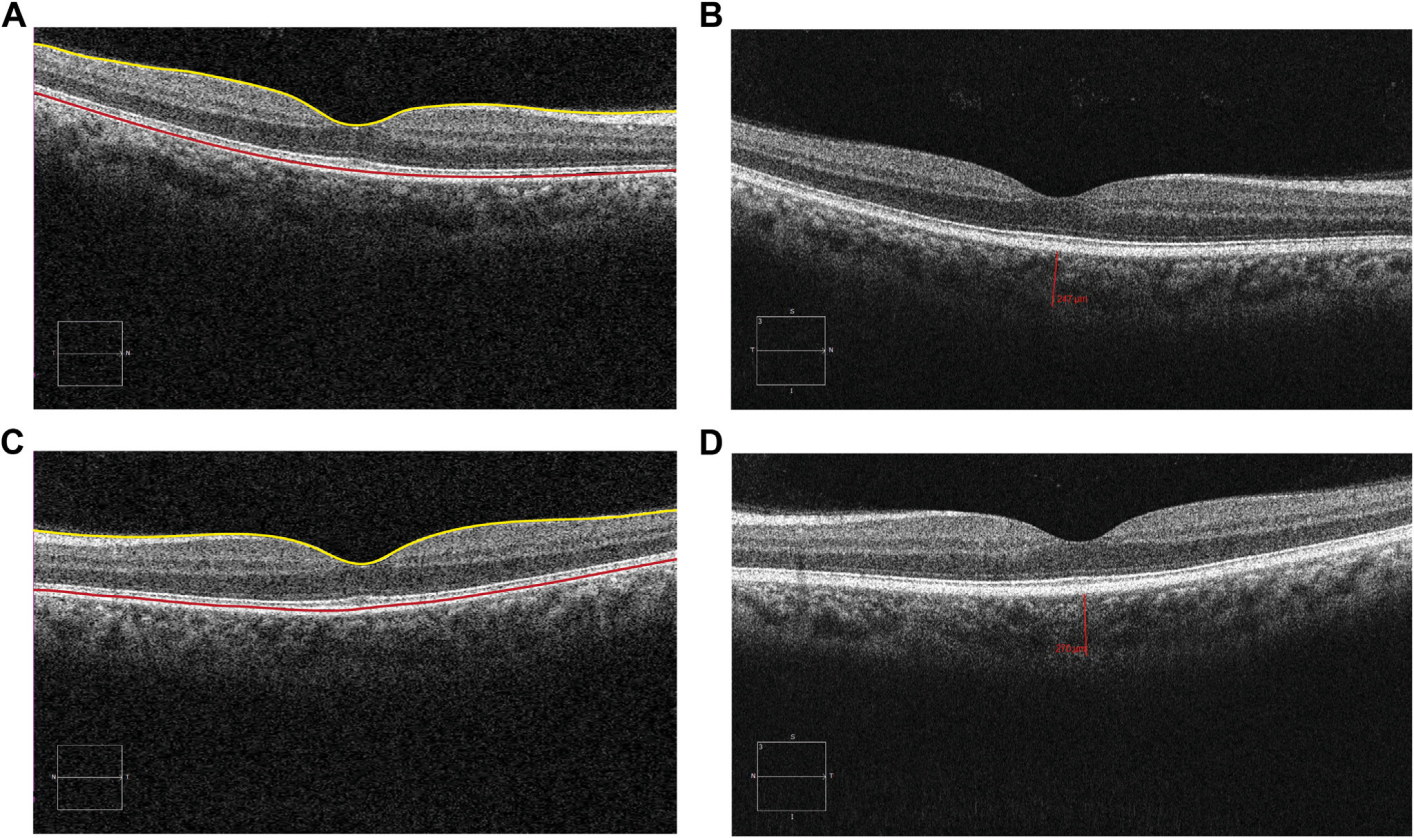
OCT scans recorded in the right (**A, B**) and left (**C, D**) eyes of a 50-year-old Ghanaian male. In (**A**) and (**C**) the segmentation of the inner limiting membrane and retinal pigment epithelium layer is shown. The quantification of the thickness of the central choroid is shown in (**B**) and (**D**).

Both macular retinal and choroidal thicknesses were measured using the Cirrus HD-OCT proprietary software.^46^ All OCT B-scans were manually inspected to ensure that the inner limiting membrane and retinal pigment epithelium layers were accurately segmented, and that the hyporeflective border between choroid and sclera was visible; see Figure 1. For the retina, the Cirrus HD-OCT software generates false color 2- and 3-dimensional thickness maps presented in 9 segments corresponding to the quadrants of the ETDRS.^47^ The inner (3 mm) and outer (6 mm) rings of the map, which corresponded to the parafoveal and perifoveal regions, respectively, were divided into superior, inferior, nasal, and temporal quadrants. The central 1 mm diameter region corresponded to the central foveal area. Central choroidal thickness was measured at the thinnest center point of the fovea manually from "5-Line Raster" high-definition images using the "Ruler" tool.^48^ These measurements were undertaken by 2 observers (J.S. and V.S.). A third observer (W.A.) adjudicated if there was any disparity (defined as a difference > 10% between observers) in measurements.

### Systematic Review

The objective of the systematic review was to determine typical retinal and choroidal thickness values among individuals with African ancestry. Searches were undertaken on PubMed, Google Scholar, and Scopus from their inception to August 31st, 2022. The terms "retina," "retinal thickness," "macular thickness," "retinal macular thickness," "choroid," "choroidal thickness," "central choroidal thickness," "subfoveal choroidal thickness," and "optical coherence tomography," were searched individually and then combined with "race," "ethnicity," "African," "Africa," or "African American." Reference lists were hand-searched and relevant citations screened for additional sources. Conference abstracts were included. Studies were eligible for inclusion if they reported measurements in any quadrant of the ETDRS map, specified the imaging modality used, and provided a mean or median and standard deviation with sample size, 95% confidence interval (CI), or standard error for each measurement. Studies that did not report information on the age of participants (mean or median) were excluded. In studies considering differences between multiple ethnic groups, only measurements and information collected among subjects with African ancestry were recorded. Within each study, if measurements were reported for both left and right eye, 1 eye was randomly selected for inclusion in the meta-analysis. Only measurements made among healthy individuals were included in the meta-analysis.

### Sensitivity to Additional Confounders

We sought to determine how large the effect of confounders not considered in our study would have to be to affect the significance of the association with ethnicity that we report. We did so using Monte Carlo simulations (n = 5.05 × 10^6^). Each simulation introduced a random error, ranging from 0% to 100% of the original measurement, to the retinal or central choroidal thickness measured in each eye. A *P* value (here denoted *P*_*sim*_) for association with ethnicity while adjusting for age and sex was computed for each simulation. The association was assumed to remain significant if the proportion of *P*_*sim*_ < 0.05 was > 95% or 99%. Convergence analyses were carried out to determine the minimum number of Monte Carlo simulations to run.

### Statistical Analyses

Statistical analyses were carried out using R (The R Foundation, software version 4.2.0).^49^ The level of significance for all tests was set at α = 0.05. All *P* values provided are 2-tailed and adjusted for multiple comparisons using Bonferroni corrections. Bland-Altman plots^50^ and Pearson’s correlation coefficient (denoted *r*) and its CI were used to identify correlations between retinal thickness measured in the left and right eyes of the same individual. Associations between ethnicity, age, sex, and retinal or choroidal thickness were assessed by using mixed-effect linear regression models implemented in the R package *lme4*.^51^ Dependencies between measurements made in the left and right eye of the same subject were accounted for by including a random effect term of the form (1 | Subject). Power calculations for multiple regressions were performed by assuming that the proportion of variance explained by ethnicity, age, and sex was between 20% and 30%.^43^ Sample sizes in this study ensured that regression models including 3 covariates (age, sex, and ethnicity) had a power > 85%. Post hoc power analyses were performed using the R package *simr*^52^ to determine the power associated with each regression model. Outputs from the mixed-effect models were visualized and plotted using the R packages *ggplot2*^53^ and *sjPlot*.^54^ Meta-analyses were performed using the R package *metafor*^55^ and random-effect linear regression models. Forest plots were used to visualize thicknesses estimated in each study identified through systematic review. Heterogeneity between published investigations was assessed using Cochran’s Q statistic^56^ and the *I*^*2*^ metric.^57^

## Results

### Patient Demographics

A total of 992 eyes of 496 subjects were examined during 3 visits to Ghana (128 individuals on the first visit, 204 on the second visit, and 164 on the third visit). Color fundus images were available from 990 (98.8%) eyes, and OCT scans were captured in 493 eyes (49.7%). OCT scans from 69 eyes (7%) were ungradable due to medial opacity. A total of 424 OCT volume scans were of sufficient quality to allow for segmentation. Of these, 78 scans captured in 42 Ghanaians met the inclusion criteria and were processed for further analyses (see Fig 1). Fundus images and OCT volume scans were available and of sufficient quality for segmentation for all eyes from the European group, which consisted of 37 individuals (see Table 1). Fifty-seven percent (27 participants) of Ghanaians were women; this proportion did not differ significantly from that of the European group (76%, 28 subjects, *P* = 0.4). The median age for all participants was 59.5 years old, with an interquartile range (IQR) of 12.5 years. The median age of Ghanaians (60, IQR 8.5) did not differ significantly from that of subjects with European ancestry (58.0, IQR 20.0, *P* = 0.7). Measurements made in the left and right eye of the same patient were dependent and strongly correlated in all regions of the retina and choroid sampled (0.60 ≤ *r* ≤ 0.95; *P* < 0.001 for all regions). Median AL among participants with European ancestry was 23.4 (IQR 1.3). Axial length was not measured among Ghanaian subjects.

**Table 1:** Baseline Demographics of Ghanaians and Participants with European Ancestry Included in This Study

Demographic	White (n = 37)	Ghanaians (n = 42)	*P* Value for Differences
Sex			
Females	28	27	0.4∗
Males	9	15	
Age			
Median (IQR)	58 (20)	60 (8.5)	0.7†
Range	41–85	45–82	-
Eyes (included in analysis)			
OD	37	40	-
OS	37	38	

IQR = interquartile range; OD = right eye; OS = left eye.

∗ Chi-square test, χ² = 0.73.

† Wilcoxon rank sum test, W = 820.5.

### Retinal Thickness among Ghanaians and Subjects with European Ancestry

**Figure 2:**
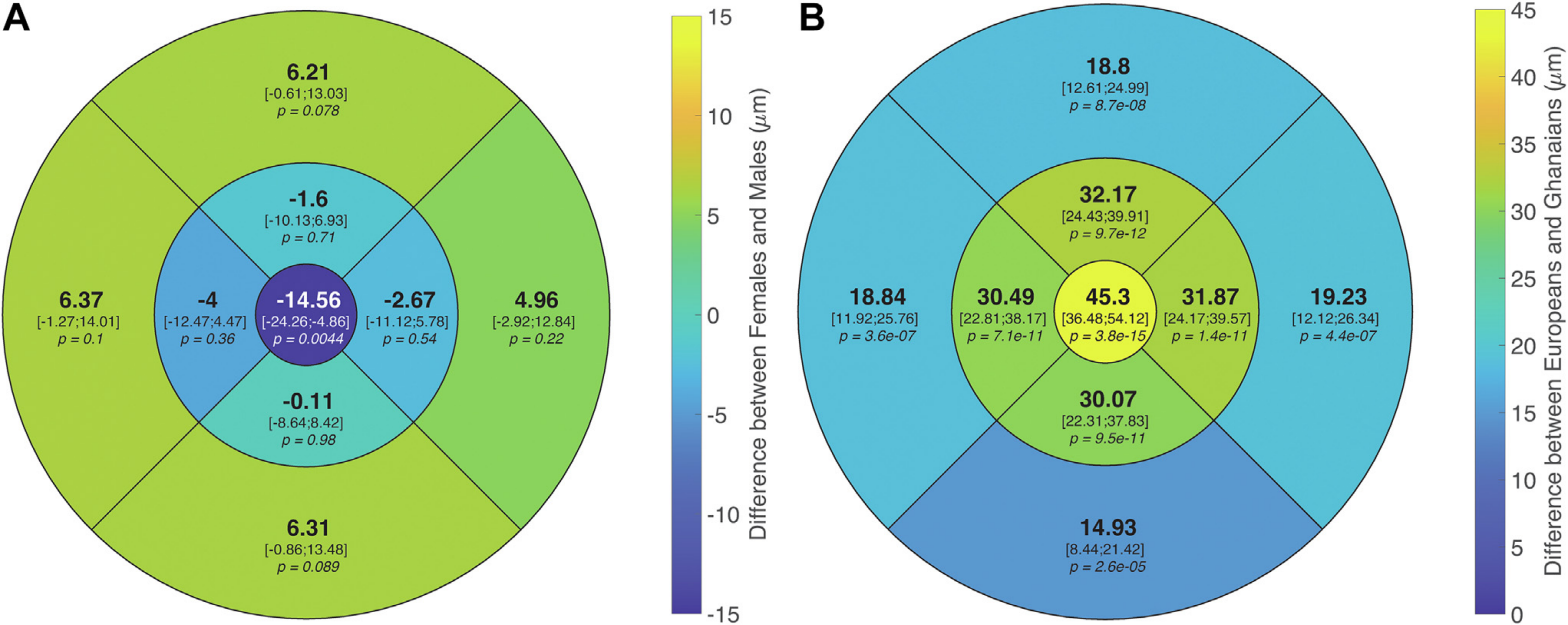
Differences in retinal thickness between (**A**) females and males and (**B**) participants with European ancestry and Ghanaians, plotted for each quadrant of the ETDRS map using the right eye as a template. In (**A**), thickness measured among females was used as a reference. In (**B**), thickness measured among the European group was used as reference. In both (**A**) and (**B**), estimates were determined using mixed-effect linear regression models generated independently for the 9 sectors of the ETDRS map and include 95% confidence intervals and associated *P* values. Estimates in (**A**) are adjusted for age and ethnicity. Estimates in (**B**) are adjusted for age and sex.

Retinal thickness was investigated using mixed-effect linear regression models including age, sex, and ethnicity as covariates. When adjusted for ethnicity and age, the retina of females was on average thinner than that of males within the parafoveal region (see Fig 2A). This difference was statistically significant in the central foveal region (*P* = 0.004). In contrast, the retina of females was on average thicker in the perifoveal region, although this difference did not reach statistical significance in any of the ETDRS quadrants (*P* > 0.07).

When adjusted for age and sex, the retina of Ghanaians was on average significantly thinner than that of participants with European ancestry in every region of the macula (*P* < 0.001; Fig 2B). The largest difference in thickness between the 2 groups was observed at the fovea (mean difference 45.3 μm, 95% CI [36.48; 54.12], *P* < 0.001). The amplitude of this difference decreased radially with distance from the foveal region, ranging from 30.07 μm (95% CI [22.31; 37.83]) to 32.17 μm (95% CI [24.43; 39.91]) in the parafovea to 14.93 μm (95% CI [8.44; 21.42]) to 19.23 μm (95% CI [12.12; 26.34]) in the perifovea.

We observed a reduction in retinal thickness with age in all regions of the retina (Fig S3) independent of sex or ethnicity. This decrease was statistically significant in the superior (*β* = −6.8μm/decade, *P* = 0.001), nasal (*β* = −5.2μm/decade, *P* = 0.01) and inferior (*β* = −4.9*μ*m/decade, *P* = 0.02) regions of the parafovea, and in the superior (*β* = −4.4μm/decade, *P* = 0.008) perifovea.

### Central Choroidal Thickness Among Ghanaians and Subjects with European Ancestry

**Figure 4:**
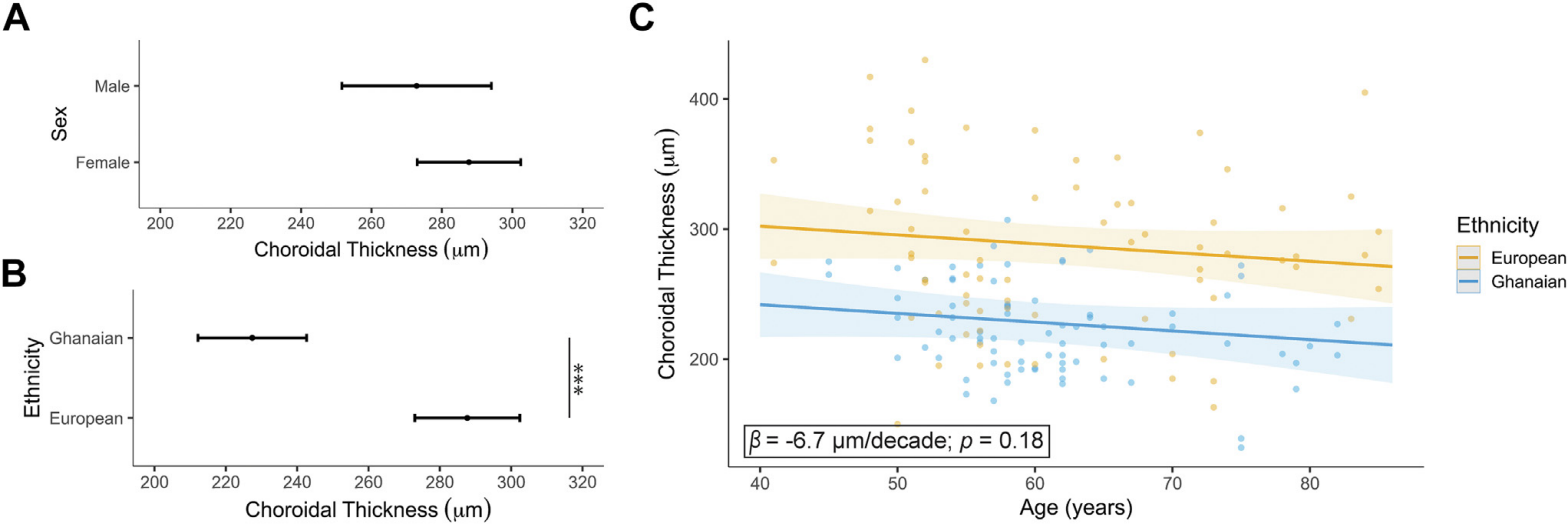
Variation of the choroidal thickness with (**A**) sex, (**B**) ethnicity, and (**C**) age. Estimates and 95% confidence intervals were determined using mixed-effect linear regression models including age, sex, and ethnicity as covariates. In (**C**), the 95% confidence interval is shown for the slope of each regression. (∗∗∗*P* < 0.001).

Central choroidal thickness was investigated using mixed-effect linear regression models including age, sex, and ethnicity as covariates. The central choroid was thinner among males compared with females (mean difference 14.87 μm, 95% CI [6.4; 36.14]); however, this difference was not significant (*P* = 0.17); see Figure 4A. The central choroid was thinner among Ghanaians compared with subjects with European ancestry (mean difference 60.28 μm, 95% CI [41.01; 79.55], *P* < 0.001); see Figure 4B. When adjusted for ethnicity and sex, age was associated with a reduction of the choroidal thickness by 6.7μm/decade on average (Fig 4C). This decrease was, however, not statistically significant (*P* = 0.18).

### Sensitivity to Additional Confounders

We sought to assess the sensitivity of the differences that we observed between Ghanaians and individuals with European ancestry to potential confounders not accounted for in our regression models. These include genetic and environmental factors, but also potential differences in ocular parameters between these 2 groups. To do so, we performed Monte Carlo simulations to simulate the effect of an added error in measurement caused by potential confounders. Each simulation introduced a random error, ranging from 0% to 100% of the original measurement, to the retinal or central choroidal thickness measured in each eye of Ghanaian participants. A *P* value (here denoted *P*_*sim*_) for association with ethnicity (adjusted for age and sex) was then computed for each simulation. Based on these analyses, we observed that the association in the temporal perifovea was the most sensitive to uncertainties in measurements. The most robust association with ethnicity was that found with central choroidal thickness. We estimated that this association remained significant (threshold for *P*_*sim*_ set at 99%) if errors in measurements remained below 52%. If the threshold for *P*_*sim*_ is set at 95%, then measurement errors would have to be smaller than 62% (see Fig S5).

### Comparison with Previous Studies

No studies assessing central choroidal thickness among subjects with African ancestry were identified through the systematic literature search. An approximate average central choroidal thickness of 375 μm was reported among a group of overseas students and foreign residents in central China (mean age 26.9).^58^ We identified 10 studies that examined variations in macular retinal thickness among individuals with African ancestry, 7 of which satisfied the inclusion criteria. Five of these studies^30^, ^31^, ^32^^,^^34^^,^^35^ were performed in the United States and reported measurements made among African Americans. Only 1 eligible study was carried out on the African continent (South Africa).^59^ Moderate to high levels of heterogeneity were observed among retinal measurements reported for African Americans in all regions of macula (52.9% ≤ I^2^ ≤ 92.5%, *P* < 0.07). To reduce this heterogeneity, we grouped studies based on the mean age of the subjects considered before generating pooled estimates. Two studies^31^^,^^35^ reported a mean age among participants lying between 20 and 30 years (21 and 28.9 years, respectively). Two investigations^30^^,^^34^ considered subjects between 40 and 50 years of age on average (47.7 and 49, respectively). The remaining study^32^ comprised individuals 30–40 years of age on average (mean 36.2); see Table S2.

**Figure 6:**
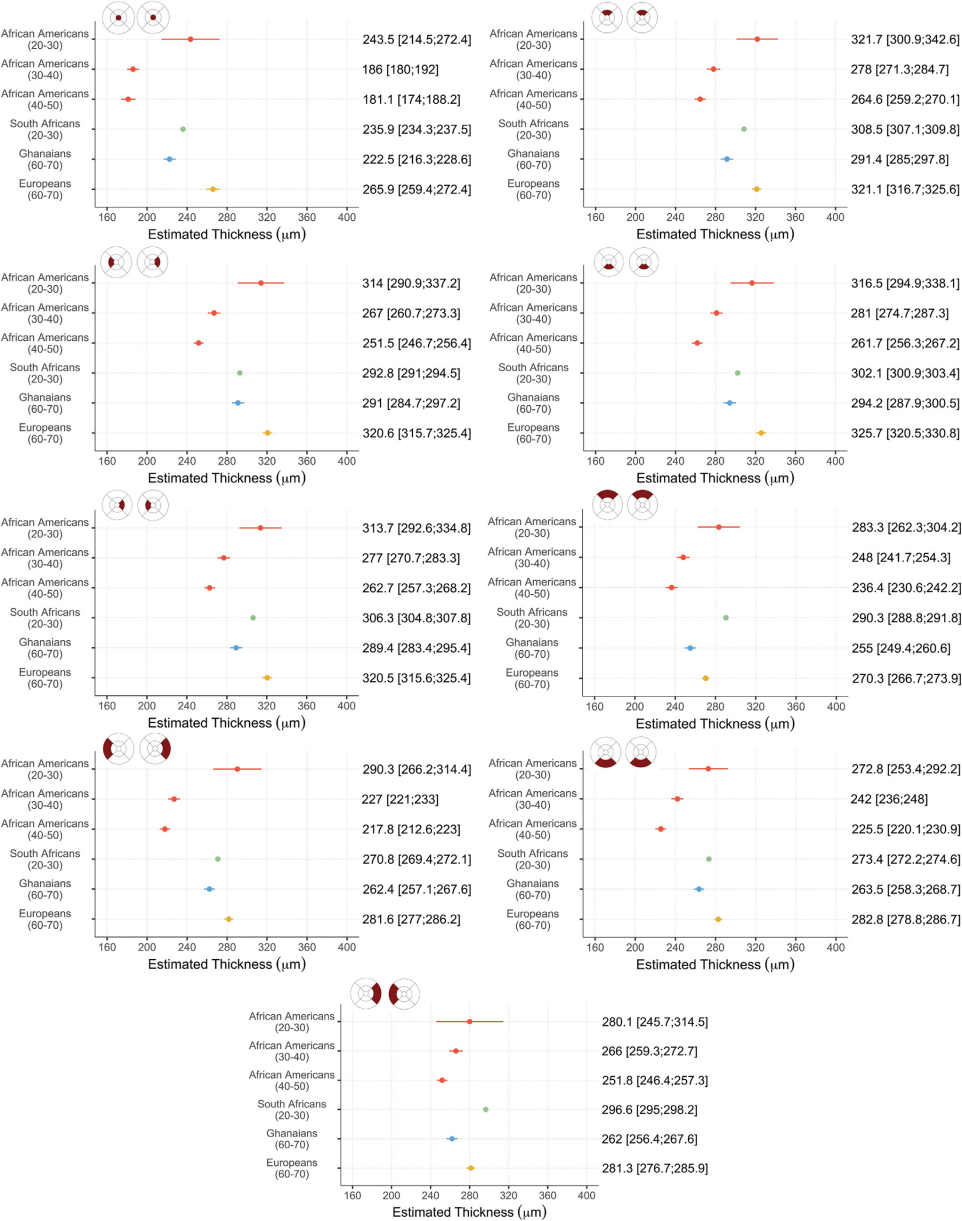
Estimates of retinal thickness among African Americans, black South Africans, Ghanaians, and a group of individuals with European ancestry, by age group, with associated 95% confidence intervals. The age group was determined based on the mean age of subjects reported by each study and is indicated between parentheses. Estimates for African Americans from the 20–30 and 40–50 age group were pooled through meta-analysis. For consistency with the methodology applied for this analysis, estimates plotted for Ghanaians and subjects of European descent are not adjusted for age or sex.

Estimates pooled among African Americans, mean retinal thickness reported among black South Africans, and those generated for Ghanaians and subjects with European ancestry considered in this study are plotted by age group in Figure 6. For consistency with the methodology applied for the meta-analysis, estimates plotted for Ghanaians and individuals of European descent were not adjusted for age or sex. We observed marked differences in retinal thickness between ethnic groups. The retina of Ghanaians was thinner than that of black South Africans in all quadrants of the ETDRS map. The amplitude of these differences ranged from 2.8 μm in the temporal parafovea to 35.3 μm in the superior perifovea. Among African Americans, we observed a reduction in pooled retinal thickness estimates with increasing age group in all regions of the macula. Retinal thickness measured among Ghanaians was thinner than that obtained from African Americans in the 20–30 age group in all ETDRS quadrants, with differences ranging from 9.3 μm in the inferior perifovea to 30.3 μm in the superior parafovea. It was on average thicker than that among African Americans from the 30–40 and 40–50 age groups. The amplitude of these differences ranged from 18.6 μm in the superior perifovea to 44.6 μm in the temporal perifovea.

## Discussion

Macular retinal thickness among Ghanaians differs markedly from that of individuals with European ancestry, African Americans, and other previously reported indigenous African populations. The retina in all regions of the macula is on average significantly thinner among Ghanaians as compared to individual of European descent. This difference is independent of age and sex; it is largest within the central 1 mm foveal region and decreases in amplitude radially with distance from the foveal center. Systematic review and meta-analyses of previous reports indicate that macular retinal thickness differs markedly between Ghanaians, black South Africans, and African Americans, and that some of these differences may be partly driven by age. Central choroidal thickness is also significantly thinner among Ghanaians as compared to subjects with European ancestry.

Previous studies have been consistent in reporting that the retina of African Americans^30^, ^31^, ^32^, ^33^, ^34^, ^35^, ^36^ was on average thinner than that of individuals with European ancestry. The reduction in pooled retinal thickness estimates with age that we observed among African Americans in all quadrants of the ETDRS map indicates that age should be carefully considered when comparing retinal features between populations. This finding is reinforced by the fact that, when compared to the group of individuals with European ancestry that we considered, macular retinal thickness was smaller among African Americans in the 30–40 years and 40–50 years age groups in all quadrants of the ETDRS map but was generally thicker in the outer quadrants among African Americans from the 20–30 years age groups. The lack of adjustment for age could explain why the mean central macular thickness among black South Africans was previously found to be higher than averages from African Americans and European subjects.^59^ A reduction in retinal thickness with age has been demonstrated among individuals with European ancestry,^60^ a pattern that we also observed among Ghanaians. A previous study performed among individuals with predominantly European ancestry found that foveal, perifoveal, and peripheral macular thicknesses decreased in patients > 60 years of age; however, it was predicted to increase with age in younger individuals in some regions of the macula.^61^ These findings highlight the need for further investigations to elucidate the contribution of age to macular retinal thickness and its differential effect on both genetically mixed and indigenous groups.

We previously reported that the foveal pit of Ghanaians was significantly wider and larger in volume as compared to that of a group composed of individuals with European ancestry.^43^ This variation may partly explain why the amplitude of the differences in retinal thickness between Ghanaians and subjects of European descent is maximal at the fovea and within the parafoveal belt of the macula and decreases radially with distance from its center. Significant differences in the morphology of the foveal depression and in their pattern of variation with age between Ghanaians and individuals with European ancestry may be important drivers of the dissimilarities in retinal thickness that we observed. Future studies will seek to determine longitudinal changes in the shape of the foveal pit and how they may influence retinal thickness among subjects with African or European ancestry. While our study measured total retinal thickness only, future studies will be directed toward investigating associations with specific retinal sublayers and their blood supply to gain a better understanding of mechanistic differences underlying ethnic differences. Retinal layers, including the outer nuclear layer, the outer plexiform layer, the inner nuclear layer, the inner plexiform layer, and the ganglion cell layer each serve a unique function in the visual process^60^^,^^62^ and may vary in their structure with ethnicity. Previously reported differences in the vascular density of the superior, intermediate, and deep retinal capillary plexuses within the fovea and parafovea among healthy subjects with African ancestry compared with subjects of European descent^63^^,^^64^ may also be key factors to consider.^60^

The central choroid was found to be significantly thinner among Ghanaians compared with participants of European descent. This finding is consistent with a previous study carried out among overseas students and foreign residents in central China, which found that the subfoveal choroid was thinner among participants of African descent compared with individuals with European ancestry.^58^ The significance of this finding is unclear. The main function of the choroid is to support the metabolic requirements of the outer retina, which includes the photoreceptor cells. The choroid plays an essential part in the delivery of oxygen and nutrients to the outer retina and the clearance of fluids and metabolism byproducts from the subretinal space.^60^^,^^65^, ^66^, ^67^ How these functions relate choroidal thickness is unknown, mainly because this feature has mainly been investigated in the context of retinal diseases. Reduced choroidal thickness has been reported in eyes presenting large drusen or reticular pseudodrusen,^68^^,^^69^ pigmentary abnormalities,^70^ and in late stage AMD (neovascular AMD and/or geographic atrophy).^24^^,^^70^ While choroidal vascularity also varies over the course of AMD^71^ and between populations with European and African ancestry,^63^^,^^64^ its association with choroidal thickness remains to be clarified.^72^

The main limitation of this study was that only a small number of confounders were considered. We evaluated the significance of this shortcoming by performing sensitivity analyses for associations between retinal or choroidal thickness and ethnicity. Our sample size was too small to effectively assess the contribution of weakly associated factors such as body mass index,^59^ lens thickness,^73^ or smoking^70^ on retinal or choroidal thickness. Axial length has been associated with thin inner and outer macula, but not central macula.^59^ While previous studies indicate that AL is unlikely to have a strong effect on retinal thickness measurements,^45^ it has been shown to be an important confounder for choroidal thickness.^58^^,^^59^^,^^70^^,^^71^ Mean AL among our European group lied within a range similar to that of participants from studies included in our meta-analyses (see Table S2). Since we did not have access to AL measurements among Ghanaians, we estimated the expected range of errors introduced by variations in AL on choroidal thickness measurements from a previous study.^58^ Based on this investigation, the largest error introduced by AL is approximately 42%, which is below the 52% error in measurements necessary to affect the significance of differences in choroidal thickness (see Fig S5). Therefore, the lack of correction for AL is unlikely to affect the observed association between choroidal thickness and ethnicity among our participants. The imbalance between the number of males and females among Ghanaians and individuals with European ancestry in our study did not allow us to effectively investigate associations between sex and retinal and choroidal thickness. Retinal thickness was on average smaller within the fovea and parafovea among females, in agreement with previous investigations,^31^^,^^34^^,^^39^^,^^59^^,^^74^ but not in the perifovea, which is inconsistent with previous reports.^35^^,^^75^, ^76^, ^77^ Future studies will include a larger number of participants and systematically investigate the effect of additional genetic and environmental confounders on retinal and choroidal parameters.
