# Supplementary material for: Retinal and Choroidal Thickness in an Indigenous Population from Ghana: Comparison with Individuals with European or African Ancestry
Source: Ophthalmol Sci. 2023 Aug 21;4(2):100386. doi: 10.1016/j.xops.2023.100386 (PMC10585639; doi:10.1016/j.xops.2023.100386)
Supplement: Table S2 [file mmc1.pdf]

Table S2 Measurements of retinal thickness made among healthy individuals with African ancestry identified through systematic review. Mean age and mean axial lengths were also included when reported.

Study (Year)	Country	Ethnicity	N	Mean Age (SD) (Age group)	Mean Axial Length (SD)	Region Sampled	Mean Thickness (SD)
**Kelty (2008)**	United States	African Americans	31	37.7 years (11.2) (30-40)	24.3 mm (0.9)		186 µm (17)
							278 µm (19)
						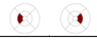	267 µm (18)
							281 µm (18)
							277 µm (18)
							248 µm (18)
						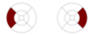	227 µm (17)
						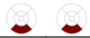	242 µm (17)
						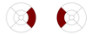	266 µm (19)
**Girkin (2010)**	United States	African Americans	620	45.1 years (13.3) (30-40)	23.70 mm (0.97)		184.4 µm (20.2)
							266.2 µm (16.8)
							253.4 µm (15.7)
							263.1 µm (16.7)
							264.7 µm (17.7)
						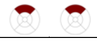	233.4 µm (15.6)
						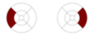	217.8 µm (15)
						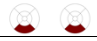	225.7 µm (15.1)
						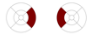	249.5 µm (17.1)
**Kashani (2010)**	United States	African Americans	42	49 years (2) (40-50)	Not Reported		181 µm (3.7)
							264.6 µm (2.8)
							251.5 µm (2.5)
							261.7 µm (2.8)
							262.7 µm (2.8)
							236.5 µm (3)
						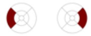	217.8 µm (2.7)
						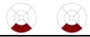	225.5 µm (2.8)
						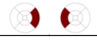	251.9 µm (2.8)
**Wagner-Schuman (2011)**	United States	African Americans	30	25.6 years (9.9) (20-30)	Right Eye 24.3 mm (0.9)<br>Left Eye 24.2 mm (0.9)		245.2 µm (21.9)
						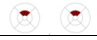	324.7 µm (14.2)
							323.4 µm (16.4)
						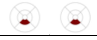	320.6 µm (15.2)
							310.1 µm (14.6)
							282.5 µm (15.2)
						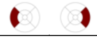	300.9 µm (11.5)
						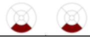	270.6 µm (14)
						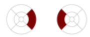	263.8 µm (14)

Table S2 *(continued)*

Study (Year)	Country	Ethnicity	N	Mean Age (SD) (Age group)	Mean Axial Length (SD)	Region Sampled	Mean Thickness (SD)
**Harb (2012)**	United States	African Americans	104	21 years (1.3) (20-30)	Not separately reported for African Americans		242 µm (20)
							318 µm (16)
						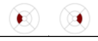	304 µm (17)
							312 µm (16)
							318 µm (16)
							284 µm (15)
						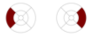	276 µm (15)
							275 µm (14)
						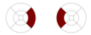	299 µm (17)
**Mashige (2017)**	South Africa	Black South Africans	600	28.15 years (13.09) (20-30)	23.05 mm (0.98)		235.89 µm (20.04)
							302.12 µm (15.63)
							292.77 µm (21.55)
							302.12 µm (15.63)
						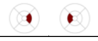	306.28 µm (18.71)
						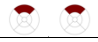	290.32 µm (18.44)
						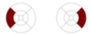	270.77 µm (17.16)
						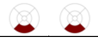	273.39 µm (15.04)
						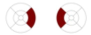	296.58 µm (20.3)
