# Supplementary material for: Retinal and Choroidal Thickness in an Indigenous Population from Ghana: Comparison with Individuals with European or African Ancestry
Source: Ophthalmol Sci. 2023 Aug 21;4(2):100386. doi: 10.1016/j.xops.2023.100386 (PMC10585639; doi:10.1016/j.xops.2023.100386)
Supplement: Figure S3 [file mmc2.pdf]

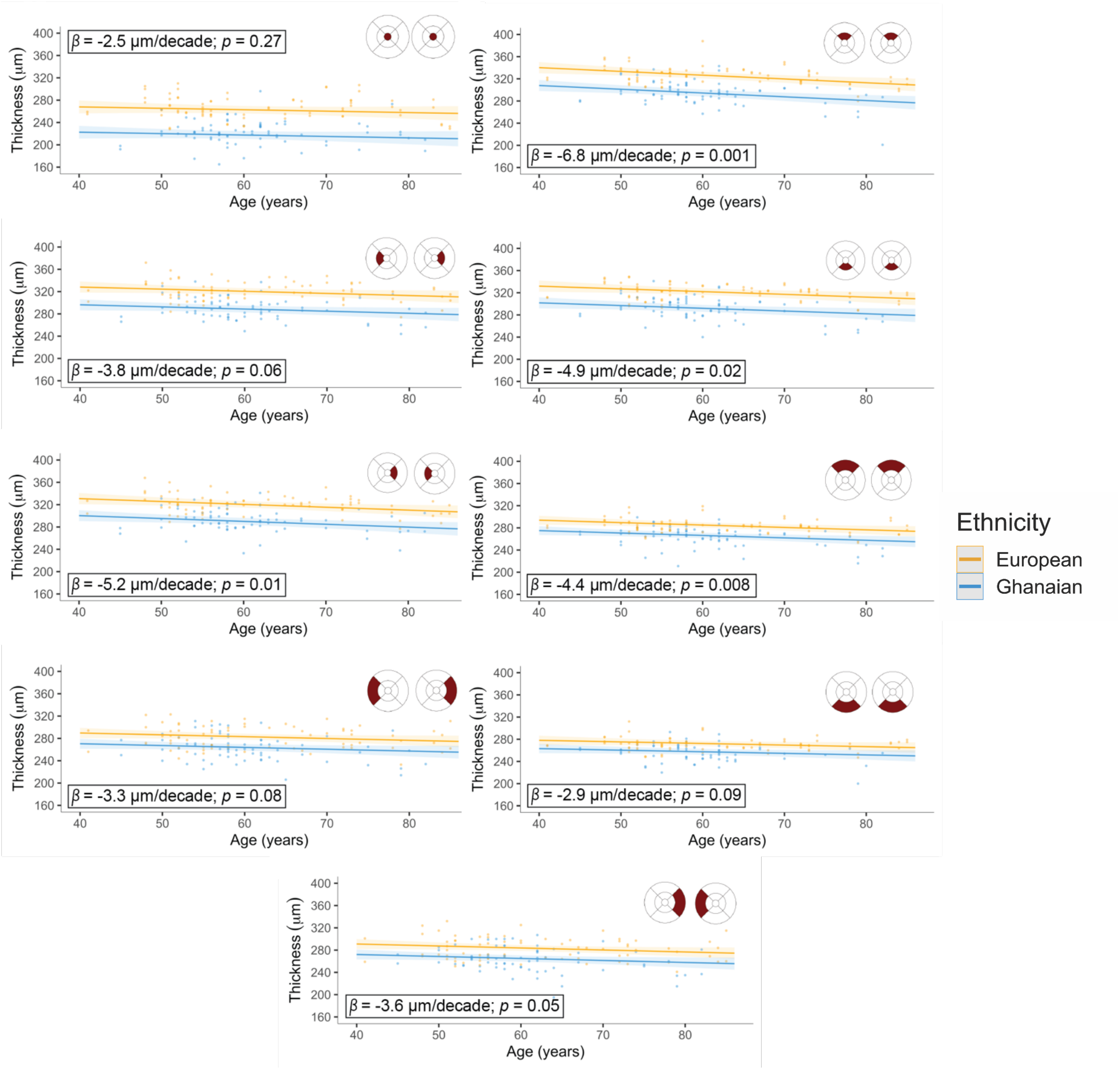

Figure S3. Variation of retinal thickness in each quadrant of the ETDRS map with age, by ethnicity. The slope was determined using mixed-effect linear regression models computed independently for the nine sectors of the ETDRS map. The 95% confidence interval is shown for the slope of each regression along with the measurements for all eyes available.
