# Supplementary material for: Retinal and Choroidal Thickness in an Indigenous Population from Ghana: Comparison with Individuals with European or African Ancestry
Source: Ophthalmol Sci. 2023 Aug 21;4(2):100386. doi: 10.1016/j.xops.2023.100386 (PMC10585639; doi:10.1016/j.xops.2023.100386)
Supplement: Figure S5 [file mmc3.pdf]

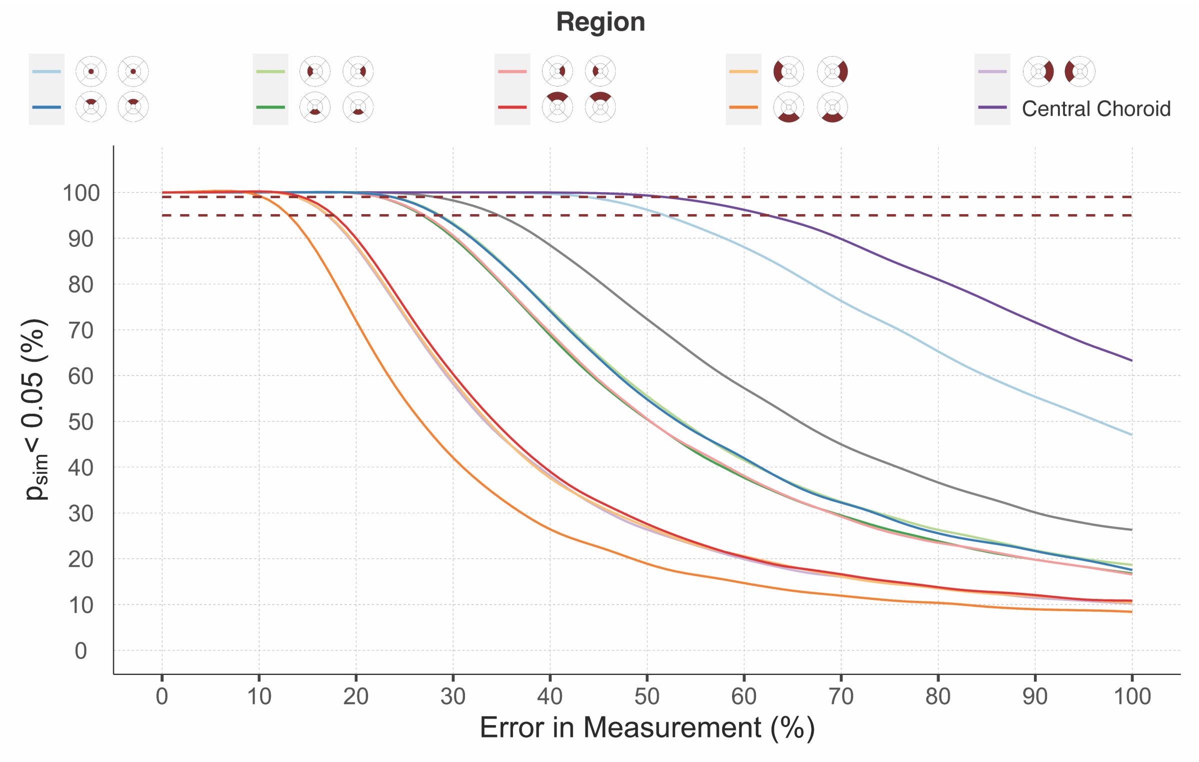

Figure S5 Sensitivity of associations between retinal or choroidal thickness and ethnicity to potential confounders not accounted for in mixed-effect linear regression models. The curves were generated by introducing a random error, ranging from 0 to 100% of the original measurement, to retinal or central choroidal thickness measured in each eye of Ghanaian participants. A *p*-value (here denoted P_{sim}) for association with ethnicity was then computed for each simulation. Estimates were determined using mixed-effect linear regression models adjusted for age and sex. The 99% and 95% thresholds are highlighted with dashed lines.
